# Unseen and unread decision letters in everyday care: a case study of medication management assistance in Norwegian municipal home healthcare

**DOI:** 10.1080/02813432.2026.2669814

**Published:** 2026-05-14

**Authors:** Marianne Kollerøs Nilsen, Hege Sletvold, Sue Jordan, Rose Mari Olsen

**Affiliations:** a Faculty of Nursing and Health Sciences, Nord University, Namsos, Norway; b Center for Care Research, Namsos, Norway; c Faculty of Nursing and Health Sciences, Nord University, Stjørdal, Norway; d Department of Nursing, College of Human and Health Sciences, Swansea University, Swansea, United Kingdom

**Keywords:** Patient safety, home healthcare, medication management, decision letters, Safety-II, qualitative research, Norway

## Abstract

**Background:**

Medication safety in home healthcare is critical for older adults with multimorbidity and polypharmacy. Norwegian municipalities allocate medication management support through legally binding individual decision letters intended to ensure transparency and guide care. Their practical role in everyday medication management remains unclear. This study explores how individual decision letters facilitate safe medication management practice in municipal home healthcare services.

**Methods:**

A qualitative multiple-case study was conducted across three rural municipalities. Data comprised 15 decision letters and 35 semi-structured interviews with nine patients, one general practitioner, four nurses, and two service allocators over 12 months. Reflexive thematic analysis was used to identify patterns in how the decision letters were interpreted and implemented in practice.

**Results:**

Decision letters were seldom read by patients or nurses and often used vague language that required interpretation. Nurses relied on supplementary structures – care plans, task lists, and verbal agreements – to operationalise medication support. Variability in interpretation and revision processes created uncertainty for patients and limited their ability to exercise their rights. Financial constraints further shaped service allocation.

**Conclusion:**

Decision letters function primarily as formal legal instruments rather than practical guides. Their limited clarity and accessibility reinforce reliance on informal systems and discretionary practices. Enhancing language precision, integrating letters with care plans, and involving patients in reassessments may improve medication safety and patient empowerment.

## Background

Patient safety is fundamental to healthcare, involving systematic efforts to build a safety-oriented culture, establish effective processes, and design environments that reduce errors and their consequences [1]. Ensuring safe medication management support for older adults living at home is an increasingly pressing concern and a critical element of nursing care [2,3]. Medication errors and unsafe practices constitute a significant source of preventable harm within global healthcare systems. Errors may occur at any stage of the medication process and are frequently linked to systemic weaknesses and human factors such as fatigue, inadequate working conditions, and staffing shortages [1].

Safety is often described as a system quality that ensures the risk of harm to individuals and the environment remains low [4,5]. The World Health Organization (WHO) defines patient safety as the prevention of errors and adverse effects in medical care [6]. Moving beyond a reactive approach focusing solely on failures, Hollnagel et al. [4] introduced the concept of *Safety II*. Rather than waiting for adverse events, the Safety-II perspective emphasises the proactive examination of everyday clinical work to understand how successful outcomes are achieved. Positive outcomes are not achieved solely through adherence to procedures; they often depend on individuals making context-sensitive adjustments. Identifying and learning from these adaptive practices is just as vital as investigating the causes of failures. Given the increasing complexity and interdependence of healthcare systems, flexible and adaptive responses to unpredictable conditions are essential. Building resilient healthcare systems requires a dual approach: preventing adverse events by analysing failures and minimising risks, viewing humans as liabilities (Safety I), while also understanding effective practices by recognising human adaptability as a resource (Safety II) [4]. Additionally, patients have a key role in maintaining patient safety in healthcare services [7,8].

In Norway and other countries with decentralised healthcare systems, municipalities are responsible for organising and delivering home healthcare, that is financed through municipal taxes, government grants, and user fees [9,10]. As the population ages, an increasing number of older individuals rely on municipal home healthcare services, including support with medication management. Medication management support (MMS) refers to practical, non-clinical assistance aimed at ensuring the safe and appropriate handling of medications in home-care settings. MMS may include a range of tasks, such as ordering, storing, preparing, dispensing, and administering medications, as well as observing patients’ responses and documenting the practice. The scope and combination of tasks vary according to each patient’s needs and capabilities. In practice, MMS commonly includes adherence aids like pill organisers or multidose drug dispensing (MDD). MDD is a system in which medications are machine-packed into unit-of-use disposable bags, with each bag containing the medications intended for a specific dosing occasion [11]. Nurses provide direct care, monitor medication adherence, and coordinate with general practitioners (GPs), pharmacists, and family caregivers. Through these coordinated activities, MMS enables individuals who have reduced capacity for self‑management to maintain safe and consistent medication use in everyday life. However, MMS may present distinct challenges. Older adults often face age-related changes, multimorbidity, and polypharmacy, which increase the risk of drug-related problems [12–14]. In this context, healthcare professionals must navigate clinical decision-making, documentation practices, and interprofessional collaboration, all within the constraints of municipal service structures and home environments. Research has shown that systemic challenges, such as fragmented communication, unclear documentation practices, and varying levels of professional competence, can compromise medication safety in home care settings [15–17].

Legally binding individual decision letters outline a person’s entitlement to services, specifying type, scope, and duration. Issued within 30 days of application, these letters guide collaborative care planning and ensure transparency [18,19]. Clear articulation of services is essential for user understanding and effective delivery [20]. Individual decision letters significantly shape the healthcare services delivered, functioning as both administrative instruments and mechanisms for translating assessed needs to care actions. While these documents typically meet legal and procedural standards, research highlights their frequent inadequacy in conveying the practical details of service provision, leading to ambiguity and implementation challenges [21–23].

This study aims to explore how individual decision letters are used to facilitate safe medication management for older adults. Adopting a Safety-II perspective [4], the study examines how safety in medication management is achieved not only by preventing errors, but also by understanding how everyday practices adapt successfully to the complex condition of home healthcare.

## Methods

### Design

This study employed a Qualitative Multiple Case Study Design [24,25], which enabled in-depth exploration of how MMS is facilitated across and within municipalities.

Case study methods are well-suited to healthcare, where complexity and contextual variation are inherent [26]. A case may be described as ‘a phenomenon occurring within a bounded context’ [24]. To prevent the scope of a case study from becoming too broad or unfocused, setting clear case boundaries is recommended [24,25,27]. In this study, a case was defined as an individual decision letter regarding MMS and how this decision was understood and enacted by the older recipient, the GP, a nurse, and the service allocator who issued the letter. Each case thus comprised the decision letter itself, complemented by interviews with the key actors involved. The study lasted one year, with follow-up interviews conducted throughout. This research design enabled a thorough investigation of the relationship between formal decision-making and everyday practices in medication management. It incorporated multiple sources of information to gather converging evidence [26].

### Study setting

Aligned with the Nordic welfare model, municipalities flexibly deliver equitable services amid rising demand and resource constraints. Many adopt a purchaser–provider split model, separating administrative and care roles. Recent reforms promote collaboration to improve care integration. Decision-making requires transparency, accountability, evidence-based justification, and inclusive stakeholder recognition, emphasising equality in healthcare resource allocation [20,28–30]. Service allocation in Norwegian municipal healthcare must be based on individual assessments and tailored to personal needs, while meeting minimum care standards.

This study involved three Norwegian municipalities. Each municipality was rural or semi-rural, had 3.4 to 38.2 citizens per square kilometer and was small to medium-sized with 4,000 to 7,000 citizens [31].

### Sampling

Sampling was purposive [32] and information-oriented [33]. A contact person from each municipality identified individuals meeting the inclusion criteria: ≥ 65 years old, living in their own homes, receiving MMS within the last four months, showing minimal cognitive impairment, and considered able to participate in an interview. In total, 14 people were asked to attend by the municipal contacts – four declined. A member of the research team (MKN) contacted ten people, and nine were recruited. The patients who declined indicated that they did not have time to participate. The nine participants agreed to be interviewed and permitted researchers to read their decision letter, as well as for GPs, nurses, and service allocators to be interviewed regarding their situation.

Nine patients, one GP, two service allocators, and four nurses participated in the study, totalling 35 interviews. The interviews lasted from four to 51 min (mean: 21 min, median: 19 min). Decision letters for eight patients were included in the analysis; one patient declined access to their decision letter. In total, 15 decision letters were included (one patient did not have a second, renewed decision letter). All service allocators and nurses were registered healthcare personnel (registered nurses, nurse assistants, or social educator), and each was responsible for more than one patient.

Except for two patients, all interviews were conducted solely between the informant and MKN. In one setting, the spouse attended, and in another, the daughter attended. An overview of informant characteristics is presented in Table 1. A full description of the cases is presented in the additional file.

**Table 1.** Informant characteristics.

	Years of age, median (min-max)	Gender, N female (%)	Educational/work background, N	Years of experience, median (min-max)
Patients n = 9	78 (65–90)	4 (44)	Healthcare background 4 Other 5	
Nurses n = 4	42 (30–50)	4 (100)	Registered Nurse 2 Nurse Assistant 2	15 (8–22)
Service allocators n = 2	(46–48)	2 (100)	Nurse 1 Social educator 1	12–30
General Practitioner n = 1		1 (100)	Specialist in general medicine	30

### Data collection

Individual interviews explore experiences and perceptions [34]. Interview guides were developed and tailored to the specific participant groups: GPs, nurses, service allocators, and patients. The interview guides were semi-structured [32], and encompassed background questions, the application process, the content of the decision letter, and the practical implementation of medication management.

**Figure 1.**
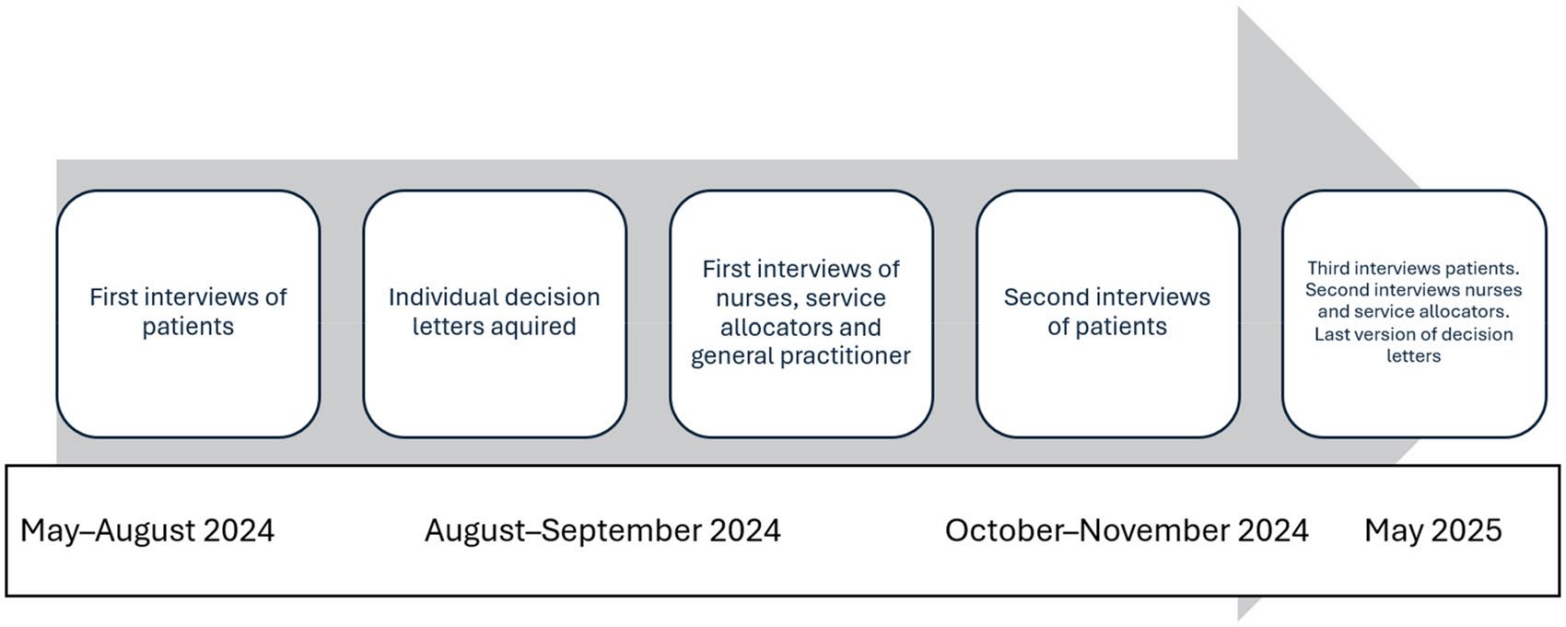
Timeline for data collection.

Over one year, MKN interviewed patients three times, service allocators and nurses twice, and the GP once at the start of the project. The individual decision letters were obtained at the beginning of the project and again one year later. The research contact obtained individual decision letters from patient records and provided MKN a paper-based copy. The interviews were conducted in Norwegian, audio recorded and transcribed verbatim; the sections reported here are translated, the first interviews with patients were face-to-face at home and all the others were digital (teams or phone). The timeline is illustrated in Figure 1.

### Data analysis

A reflexive thematic analysis was undertaken [35,36] which is well-suited to do case study research for identifying and reporting patterns (themes) within qualitative data [25,37]. We followed the six-phase approach outlined by Braun and Clarke [36]. First, MKN, RMO and HS familiarised themselves with the data by reading the interview transcripts and decision letters multiple times. Second, we generated initial codes to capture meaningful features of the dataset, involving both semantic and latent approaches. To develop themes that were both relevant to the study’s aim and grounded in participants’ narratives, an inductive approach—focused on the meanings expressed by participants—was primarily applied during coding, supplemented by deductive elements to ensure alignment with the aim. The Safety‑II perspective informed this phase by sensitising us to descriptions of successful adaptations, everyday performance variability, and instances where things went well. In the third phase, we developed initial themes by collating related codes into preliminary patterns of meaning, followed by a thorough review and refinement of these themes in the fourth phase. In the fifth phase, we defined and named the final themes to ensure clarity and coherence. Finally, we created the analytical text, integrating illustrative quotes to support and exemplify each theme.

To uphold the trustworthiness and rigor of the analytical process [38], MKN, HS and RMO were actively engaged in all phases of the analysis. Data from three categories of healthcare professionals and patients were triangulated [39] to explore interpretations of medication management strategies. Member checking further enhanced the trustworthiness of the findings [38]. All nurses and service allocators reviewed and approved the interview transcripts, and the results were discussed with them during a dissertation seminar. The findings were considered accurate, and no revisions were requested. For patient participants, repeated interviews served as a form of member checking; summaries of prior interviews were reviewed and discussed to ensure concordance in interpretation between the interviewer (MKN) and the participants. The COREQ checklist [40] guided the reporting process to ensure clarity, transparency, and comprehensive documentation of the study.

### Research ethics

The project’s procedures for managing personal data were evaluated and formally reported to Sikt – the Norwegian Agency for Shared Services in Education and Research (Ref. 838537) on 12 February 2024. All stages of the research were carried out in compliance with established ethical and regulatory standards for data protection in research. The study was conducted in accordance with the Declaration of Helsinki [41], and all participants provided informed, voluntary, written consent.

Given the limited number of cases, and to safeguard participant confidentiality, quotations are not attributed to specific individuals or cases. Instead, citations are presented in a generalised manner, identified only as ‘patient’, ‘nurse’, or ‘service allocator’ without specifying the case. None of the patients were informed about the identities of the other patients included in the study.

## Results

The analysis generated four themes: *Underutilising decision letters as information sources; Compensating for operational gaps in decision letters; Interpreting and translating decision letters into practice;* and *Navigating inconsistent revisions of decision letters.*

### Underutilising decision letters as information sources

Decision letters were not often read by either patients or nurses. Despite their formal and legal significance, these documents appear to have limited visibility and relevance in everyday care. One provider stated, ‘*I think the decision letter mainly is for the patient, whereby the patient is informed of which services are allocated.*’ A service allocator said, ‘*Few nurses actually read the decision letter, although it is intended to align with practice, right?*’ The GP said, ‘*I have to be honest, I don’t remember seeing any decision letters*.’

With few exceptions, patients reported not having read the decision letter. When asked directly, one patient responded, ‘*No, I haven’t seen any decision letter*.’ Another patient said, ‘*Well, my son has stored it in the document folder, but I’m not sure exactly where. It’s somewhere among the other papers*.’ In the second interview, a third patient reported not having read the decision letter, despite being encouraged to do so during the first interview: ‘*No, I have actually not read it*.’ Also, the letters were not readily accessible, and patients were often unsure whether they had been delivered by post or *via* a digital platform.

These statements suggest that decision letters are largely bypassed and not actively consulted by the very people they are intended to inform. The decision letter’s communicative function and accessibility for both patients and nurses are scarce.

### Compensating for operational gaps in decision letters

This theme captures how nurses compensate for the operational gaps in decision letters by creating additional structures that translate broad entitlements into actionable care. Because the decision letters often lacked the level of detail needed for implementation, nurses relied on care plans, task lists, and verbal agreements to clarify what MMS should look like in practice. As one nurse explained, care plans offered the level of detail needed for safe and consistent service delivery:

*What the patient is supposed to get help with is described much more thoroughly in the care plan. It might say, for example, that the medication is in the cupboard next to the stove, or… those small practical things. So it’s set up so that anyone here can take that task list and carry out the job and give the help the patient has a decision for*.

Service allocators also distinguished between the formal decision and the practical guidance needed for implementation: ‘*While the decision letter outlines what the patient is entitled to, the care plan provides guidance on how they* [the nurses] *should implement that entitlement*.’ However, these care plans were not routinely shared with patients or relatives.

Nurses and service allocators were aware that the description of MMS in the decision letters was often vague and lacked operational detail. One service allocator reflected on the rationale for this ambiguity:

*The decision is perhaps a bit more diffuse or softened, because we want to avoid issuing new decisions all the time. For example, the care plan might specify that the person receives visits three times a day for medication follow-up, whereas the decision letter simply states assistance with taking medications as prescribed by the doctor. This way, if the need changes—say, to only mornings—I don’t have to issue a new decision. So I don’t write in the decision letters that you get help three times a day or twice*.

However, the same service allocator acknowledged that this flexibility could create uncertainty for patients and their families:

S*ome people have expressed frustration about this, and family members might ask, ‘I want to know how often you’re visiting my mother.’ I completely understand that concern. However, if I include too much detail, I may need to revise the decision letter every week, which would require frequent reissuing*.

Overall, the theme illustrates a tension between flexibility (valued by nurses and service allocators) and predictability (valued by patients and relatives). Nurses’ use of supplementary structures reflects a form of resilience—creating clarity and continuity where the formal system leaves gaps.

### Interpreting and translating decision letters into practice

Decision letters often contained broad or standardised formulations that required substantial interpretation before they could guide practice. The phrasing used – such as *‘administer medications,’ ‘control medications,’* or *‘give medications as prescribed’* – is open to multiple interpretations and may vary depending on the context and the individuals involved. When asked to clarify what such phrases entail, service allocators and nurses offered differing explanations. This is illustrated in two quotations; One nurse’s explanation was ‘*Give medication as prescribed? Yes, that’s what the doctor prescribed (…) That’s what’s on the medication list, which we base the service on*.’ A service allocator described her understanding:

*That’s what the follow-up of the multidose drug dispensing (MDD) is. What happens, technically, is that we receive the MDD bags at the office. When it says ‘control and administer’, (…) we receive the MDD bag and compare it with the doctor’s prescription and check that the pharmacy has delivered the correct medications, that it matches our system and list, (.), then it’s delivered to the patient. So, this thing with controlling and administrating is actually directed towards the pharmacy. (…) If it is important for us to know that the patient is taking it* [the medication] *at the right time, then it would say ‘give medication’ (…) When it says ‘give medication as prescribed,’ we visit at the times listed on the MDD bag. (…) Then we also follow up to make sure it’s actually being taken*.

Among patients with this description in their decision letters, support ranged from fortnightly visits to deliver MDD, to daily support of medication management. This variability in interpretation highlights the challenges of relying on standardised language in decision letters to communicate specific expectations.

In one case, a patient complained in both the first and second interviews that she could not open the MDD bags without the tablets falling out and preferred using a pill organiser. By the third interview, she reported having spoken with the head of home healthcare services and receiving a different type of MDD bag that she could now open. Her decision letter stated she should receive ‘*ordering medications and prescriptions, storing medications, ordering and controlling medications, and weekly dispensing of a pill organiser*.’ This wording was identical in both the original and revised letters. However, according to both the patient and nurse, the actual MMS was MDD delivered every fortnight. Although patients have the right to file complaints about the services they receive, none of the patients in this study did so.

When decision letters allow for broad interpretation, it becomes difficult for patients to understand which aspects of the MMS they can question or complain about. The content is often unclear to nurses as well, likely contributing to patients’ confusion. This suggests that decision letters do not serve as clear or consistent directives but rather require translation into care through relational knowledge and situational judgment to compensate for underspecified descriptions.

Several decision letters included the phrase, *‘You meet the criteria for receiving help in the form of municipal home healthcare services.’* However, none of the nurses or service allocators could specify what these criteria were.

### Navigating inconsistent revisions of decision letters

Decision letters formally define the scope and duration of services, and their revision is a mechanism for adapting care to patients’ needs, prompting regular evaluations. However, the process of revising varies across municipalities and can be shaped by administrative routines, practical considerations, and resource constraints. For example, revising services is simpler at the letter’s expiration, but more complex mid-term. In some municipalities, decision letters are systematically reviewed at fixed intervals, whereas in others, revisions occur only when significant changes in patients’ condition and needs are observed.

As one service allocator noted, short-term decisions made adjustments easier, while revising active decisions was more complicated. Expiration dates encourage nurses to reassess patient needs, something that might not happen otherwise. A service allocator said:

*No decision letter in our municipality lasts longer than six months, so they are reviewed regularly. For new users, the duration may be even shorter, since we don’t yet know whether the service will need to be increased or reduced. (…) It’s easier to reduce services with a short-term decision duration than to revise an active one. Revising a decision letter is a more complex process than simply letting it expire.*

While routines for reassessment varied, services were not discontinued without informing the patient. Expiration dates thus serve as a prompt for reassessing patients’ needs – something that may not otherwise occur. Typically, service allocators initiate service evaluations, often based on documentation or nurse input, but without direct patient or GP involvement. As one nurse noted:

*We are not really involved in revising it* [the decision letter], *(…) they* [the service allocators] *see the changes that have been made in practice in the documentation system, and revise the decision accordingly*.

During the 12-month study period, financial constraints intensified across all three municipalities. Both nurses and service allocators said allocating services was stricter, and patients receiving only MDD were often expected to be discharged from home healthcare services. As one service allocator explained:

*I’ve considered removing MDD from our services for years. It’s seen as necessary healthcare, but patients can arrange it privately. MDD requires a lot of follow-up, and with delivery only every other week, there’s little basis for clinical assessment.*

Among the individual cases in this study, six patients received only MDD. Some had previously received broader support following hospital discharge but had since regained independence. For example, in Case 2, the decision letter evolved over the course of a year from ‘*dose and control medications, give medications as prescribed*’ (first decision letter) to ‘*control and administer MDD* (second decision letter, expired three months ago). The patient and nurse described the same service in all interviews: fortnightly MDD delivery. The service allocator believed the service had ended, but records showed the patient still had an active MDD decision. The patient reflected, ‘*I’ve wondered if it’s really necessary for them to come, but it feels safe to have their follow-up.*’

## Discussion

The findings reveal a complex interplay among formal documentation, adaptive practices, and patient experiences in facilitating safe medication management.

### Disconnect between formal decision letters and everyday practice

Our findings reveal a significant disconnect between formal decision letters and the everyday realities of medication management in municipal home healthcare services. These practices can be understood as potential contributors to resilient care, insofar as they help bridge the operational gaps left by the decision letter. However, whether they enhance resilience depends on how well they support safe medication management across varying contexts, and on whether they mitigate or inadvertently reinforce the limitations of the formal system. At the same time, the considerable heterogeneity among Norwegian municipalities [31] implies that practices concerning decision letters inevitably require local adaptation. This variability underscores the tension between the need for flexibility in service delivery and the need for predictable, equitable, and safe medication management.

From a Safety-II perspective [4], such adaptations may be understood as part of how resilience can emerge in everyday work, as nurses anticipate, respond to, and adjust to the variability created by underspecified directives. These adjustments exemplify the common gap between ‘work-as-imagined’ and ‘work-as-done’, where formalized documents do not fully capture the complexity of home-care practice. In such situations, nurses often rely on informal routines and work-arounds to maintain safe practice. Workarounds are widely described in healthcare and medication-related processes as pragmatic response to system constraints, to protect patients and maintain continuity of care [42]. Our findings suggest that the ambiguity in decision letters serves both as a source of necessary flexibility and as a trigger for such work-around practices, illustrating how resilience is enacted in gaps between policy and practice. Taken together, these findings illustrate how safe medication management is achieved not through the decision letters alone, but through the adaptive practices that compensate for their limitations.

### Ambiguity in language – the need for interpretation

Building on the disconnect described above, the ambiguity and lack of detail in decision letters required nurses to interpret them into meaningful, actionable care in practice. This illustrates the limitations of standardised language in capturing the complexity of home-care contexts and reinforces the gap between work-as-imagined and work-as-done, a central concern in Safety-II thinking [4].

Our findings suggest that interpretation of decision letters often relied on relational continuity between nurses and patients. This concept—defined as ongoing familiarity that enables context-specific understanding—is well established in the literature [44–47]. In the participating small municipalities, this relational knowledge was feasible and supported safe care, whereas it may be harder to achieve and sustain in larger urban, high-turnover settings.

Nurses and service allocators sometimes understood the same decision letter differently. This is consistent with previous research showing that competence, experience, and contextual insight shape decision-making in medication management [16,48–51].

Health literacy plays a crucial role in the ability to comprehend information [43]. Older people often report low health literacy regarding medicines [44,45], and nurses’ competencies in health literacy and ability to recognise and address this varies [46]. When written communications, such as decision letters, are ambiguous, differences in understanding among patients and professionals carry heightened risk. Although health literacy and related factors such as digital literacy were not a focus of this study, the finding that some patients did not read or remember their decision letters suggests that broader issues of accessibility may influence how older adults engage with written information. Verbal clarification is therefore essential when decision letters do not clearly convey the intended service. Further research may examine how different communication formats affect patients’ ability to understand decision letters in home-care contexts.

### Compromising patients’ rights

Building on the interpretive challenges, our findings indicate that decision letters—although intended as formal legal instruments informing patients of their rights and entitlements [9,19,47] – are rarely read or actively consulted. Many patients were unaware of the letter’s existence or unable to locate it, while nurses described relying primarily on care plans and task lists rather than the decision letters. This limited engagement weakens the decision letter’s communicative function and reduces the patient’s ability to understand, question, or appeal the services they receive. Older patients are also perceived as a quiet and compliant generation, with a tendency to express gratitude rather than assert their rights [48].

From a legal standpoint, this challenges the principle of due process in welfare services, which requires that individuals be adequately informed to exercise their rights [47]. It also has implications for patient involvement and medication safety, both central elements in patient safety initiatives [1,49]. When the decision letters are vague – and when they are not read – the patient’s ability to assess whether the care provided aligns with the formal decision is significantly weakened. This limits meaningful involvement: patients who are unaware of the formal basis for their services have little opportunity to influence or respond to care decisions. In theory, the decision letter should empower patients; in practice, it appears to be sidelined. Prior research has similarly questioned whether user involvement in the health and care sector constitutes genuine participation, as systems designed to facilitate user involvement are not necessarily adapted to the individual service recipient [48,50].

The process of revising decision letters varied across municipalities, shaped by administrative routines and resource constraints, as in Holm et al.’s study [22]. Expiry dates often served as prompts for reassessment, but mid-term revisions were more complex and less frequent. Importantly, patients were rarely involved in these evaluations. An investigation conducted by the Office of the Auditor General of Norway in 2018 [51] revealed significant shortcomings in the allocation of health and care services to older adults. The findings indicated that services were often assigned without sufficient assessment of the individuals’ needs and preferences. In numerous cases reviewed, critical information regarding requirements for assistance was either incomplete or absent. Moreover, older adults were inadequately involved in the decision-making process prior to the municipality’s determination of service provision. Administrative decisions concerning municipal home healthcare services frequently lacked transparency, offering limited insight into the municipality’s evaluative criteria and the nature of the support to be provided. Additionally, the procedural safeguards related to the right to appeal were found to be insufficiently upheld [51]. These findings were echoed in our study, suggesting that little has changed.

Financial pressures influenced service allocation, with stricter criteria applied to patients receiving only MDD. The results of this study suggest that municipal budgets became increasingly constrained during the study period, prompting service allocators to actively seek cost-saving measures. Initially, municipalities transitioned from using pill organisers to MDD systems, citing improvements in safety and efficiency [11,52,53]. Subsequently, it was proposed that patients receiving only MDD, and no additional home healthcare services, could order their medications directly from the pharmacy. In contrast to Norway, MDD is typically provided by pharmacies in other countries, rather than through home healthcare services [54]. This shift may effectively transfer costs from the municipality to the patient. Notably, the patient perspective was absent from discussions surrounding these changes.

These findings suggest that while decision letters formally define service scope, their revision and implementation are subject to systemic variability and may not reflect patients’ evolving needs.

### Strengths and limitations

To our knowledge, few triangulated longitudinal studies have examined domiciliary medicines management and decision letters among older adults, underscoring the novelty of our findings. Transferability [38] has been addressed through purposive sampling and the provision of thick descriptions; nevertheless, a notable limitation is that the study was conducted solely within three small Norwegian municipalities.

Although six of the nine patients received only MDD, this group represents a substantial proportion of home‑dwelling older adults in Norwegian municipalities, many of whom are otherwise independent but require support with medication management. Some had previously received broader services following hospital discharge, illustrating that service needs fluctuate over time. While this limits the transferability of the findings to patients with more complex or long‑term care needs, it also reflects a common and relevant user group for whom decision letters are routinely issued.

Selection bias can arise in qualitative studies [55]. Our patient cohort comprised volunteers who were among the least ill in the home care population. Despite this, participants represented the full range of socioeconomic backgrounds in the study areas. However, social conditions in municipalities were relatively uniform. We were unable to assess for social desirability bias [56]; some participants may have been hesitant to express criticism due to concerns that their responses could be shared with healthcare nurses, although this was not explicitly stated.

## Conclusion and implications

Adopting a Safety-II perspective shifts the focus from ineffective to effective healthcare and how healthcare professionals and systems adapt and succeed in dynamic, real-world conditions. In this context, exploring individual decision letters as tools for facilitating safe medication management offers a novel and insightful angle.

Our findings suggest that the limited use and vague content of decision letters reinforce a reliance on informal systems and discretionary practices. While these adaptive practices help maintain safety and flexibility, they also expose structural vulnerabilities – particularly in terms of legal accountability and patient empowerment. Addressing this disconnect may require rethinking how formal decisions are communicated and operationalised, ensuring that legal documentation supports rather than constrains resilient and transparent care.

To translate these insights into practice, we propose the following recommendations:

1. Use consistent, patient-friendly language and include operational details (e.g. visit frequency, medication support) to reduce ambiguity. Ensure accessibility in digital and physical formats.
2. Align decision letters with care plans and task lists. Share care plans proactively with patients. Encourage dialogue among service allocators, nurses, and patients to clarify decision content.
3. Involve patients and GPs in evaluations and document the rationale for any changes to ensure accountability and continuity of care.
4. Acknowledge the critical role of frontline staff in ensuring safe medication management through adaptive practices. Promote reflective practice by creating spaces for staff to share and learn from successful adaptations.

Clarifying the role of decision letters is crucial: are they intended solely as formal legal communication, or should they also inform and guide practice for both patients and nurses, as envisioned in the legislation? Our study challenges the communicative function of decision letters and suggests that, in their current form, they are not effective tools for informing or empowering patients. Strengthening their clarity, relevance, and integration into care processes may enhance both medication safety and patient involvement in home healthcare.

## Supplementary Material

COREQ_Checklist.docx

Appendix Case overview.docx

## Data Availability

The datasets generated and analyzed during the current study are not publicly available, as participants did not consent to data sharing. However, aggregated and analyzed data are available from the corresponding author upon reasonable request (in Norwegian only).

## References

[1] World Health Organization. Medication Without Harm- Global Patient Safety Challenge on Medication Safety. Geneva: World Health Organization; 2017.

[2] Vaismoradi M, Lillo Crespo M, Turjamaa R. Nurse-led medication management for older people in home care: a systematic review of evolving nurse responsibilities in technology-assisted care. Home Health Care Management & Practice. 2025;37(2):140–153. doi: 10.1177/10848223241283415.

[3] Hellzén O, Ness TM, Ingstad K, et al. Adapting to home care in Norway: a longitudinal case study of older adults’ experiences. 2024. 10.1016/j.jaging.2024.101215 38458722

[4] Hollnagel E, Wears RL, Braithwaite J. From Safety-I to Safety-II: a white paper. The Resilient Health Care Net: published Simultaneously by the University of Southern Denmark, University of Florida, USA, and Macquarie University, Australia. 2015; https://www.england.nhs.uk/signuptosafety/wp-content/uploads/sites/16/2015/10/safety-1-safety-2-whte-papr.pdf.

[5] Reason J. Human error: models and management. BMJ. 2000;320(7237):768–770. doi: 10.1136/bmj.320.7237.768. 10720363 PMC1117770

[6] World Health Organization. Patient safety. 2020. https://www.who.int/health-topics/patient-safety#tab=tab_1.

[7] Longtin Y, Sax H, Leape LL, et al. Patient participation: current knowledge and applicability to patient safety. Mayo Clin Proc. 2010;85(1):53–62. Elsevier doi: 10.4065/mcp.2009.0248. 20042562 PMC2800278

[8] Vaismoradi M, Jordan S, Kangasniemi M. Patient participation in patient safety and nursing input–a systematic review. J Clin Nurs. 2015;24(5-6):627–639. doi: 10.1111/jocn.12664. 25178172

[9] Lovdata. Helse- og omsorgstjenesteloven. Lov om kommunale helse- og omsorgstjenester m.m. [The act on municipal healthcare services]. LOV202206-24-30:; 2011.

[10] Saunes IS, Karanikolos M, Sagan A. Norway: health system review. Health Syst Transit. 2020;22(1):1–163. 32863241

[11] Jøsendal A, Bergmo T, Granås A. Multidose drug dispensing in primary care. In Medication Safety in Municipal Health and Care Services, edited by Olsen RM, Sletvold H: Cappelen Damm Akademiske; 2022.

[12] World Health Organization. Medication safety in polypharmacy: technical report. Geneva: World Health Organization; 2019.

[13] Plácido AI, Herdeiro MT, Morgado M, et al. Drug-related problems in home-dwelling older adults: a systematic review. Clin Ther. 2020;42(4):559–572.e14. e514. doi: 10.1016/j.clinthera.2020.02.005. 32147147

[14] Woolford SJ, Aggarwal P, Sheikh CJ, et al. Frailty, multimorbidity and polypharmacy. Medicine (Baltimore). 2021;49(3):166–172. doi: 10.1016/j.mpmed.2020.12.010.

[15] Shahrestanaki SK, Rafii F, Najafi Ghezeljeh T, et al. Patient safety in home health care: a grounded theory study. BMC Health Serv Res. 2023;23(1):467. doi: 10.1186/s12913-023-09458-9. 37165357 PMC10171141

[16] Nilsen MK, Sletvold H, Olsen RM. ‘To give or not to give medication, that is the question.’Healthcare personnel’s perceptions of factors affecting pro re nata medication in sheltered housing for older adults—a focus-group interview study. BMC Health Serv Res. 2020;20(1):622. doi: 10.1186/s12913-020-05439-4. 32641030 PMC7346517

[17] Øfsti R, Devik SA, Enmarker I, et al. Compliance between registered nurses’ clinical judgment and documentation in homecare for older patients with COPD: a multiple case study. Nord J Nurs Res. 2023;43(1):1–8. doi: 10.1177/20571585221149865.

[18] Forvaltningsloven. Lov om behandlingsmåten i forvaltningssaker [Act relating to procedure in cases concerning the public administration]. LOV-1967-02-10: Lovdata; 1967.

[19] Helsedirektoratet. Veileder for saksbehandling – Tjenester etter helse- og omsorgstjenesteloven. [ Guide to case processing – Services under the Health and Care Services Act; Oslo; 2017.

[20] Pedersen AKB, Skinner MS, Sogstad M. Needs assessment in long-term care: expression of national principles for priority setting in service allocation. BMC Health Serv Res. 2024;24(1):530. doi: 10.1186/s12913-024-10889-1. 38671489 PMC11046954

[21] Ekenes M, Oldeide O, Wehling E. Allocating municipal services to individuals with complex rehabilitation needs–a discourse analysis of individual administrative decision letters. BMC Health Serv Res. 2024;24(1):460. doi: 10.1186/s12913-024-10972-7. 38609916 PMC11015684

[22] Holm SG, Mathisen TA, Sæterstrand TM, et al. Allocation of home care services by municipalities in Norway: a document analysis. BMC Health Serv Res. 2017;17(1):673. doi: 10.1186/s12913-017-2623-3. 28938892 PMC5610450

[23] Øydgard G. Individuelle behovsvurderinger eller standardiserte tjenestetilbud? En institusjonell etnografi om kommunale saksbehandleres oversettelse fra behov til vedtak [Individual needs assessments or standardized service offerings? An institutional ethnography of municipal caseworkers’ translation from needs to decisions]. TFO. 2018;4(1):27–39. doi: 10.18261/issn.2387-5984-2018-01-04.

[24] Baxter P, Jack S. Qualitative case study methodology: study design and implementation for novice researchers. The Qualitative Report. 2008;13(4):544–559.

[25] Yin RK. Case study research: design and methods. vol. 5: Thousand Oaks: Sage; 2009.

[26] Yin RK. Enhancing the quality of case studies in health services research. Health Serv Res. 1999;34(5 Pt 2)2:1209–1224. 10591280 PMC1089060

[27] Stake RE. Multiple case study analysis. New York: Guilford Press; 2013.

[28] Olver I, Dodds S, Kenner J, Health AHECotN, Council MR., et al. Ethical considerations relating to healthcare resource allocation decisions. Intern Med J. 2019;49(11):1364–1367. doi: 10.1111/imj.14461. 31713342

[29] Tynkkynen L-K, Keskimäki I, Lehto J. Purchaser–provider splits in health care—the case of Finland. Health Policy (New York). 2013;111(3):221–225. doi: 10.1016/j.healthpol.2013.05.012. 23790264

[30] Vabo SI. Velferdens organisering—mellom styring, ledelse og læring. In Velferdens organisering. edn. Edited by Vabø M, Vabo SI. Oslo: Universitetsforlaget; 2014.

[31] Kringlebotten M, Langørgen A. Gruppering av kommuner etter folkemengde og økonomiske rammebetingelser 2020 [Grouping of municipalities by population and economic framework conditions 2020]. Oslo: Statistisk sentralbyrå; 2020.

[32] Patton MQ. Qualitative research & evaluation methods. 4th ed. Thousand Oaks, London, New Dehli: Sage Publications; 2015.

[33] Flyvbjerg B. Case study. In The Sage handbook of qualitative research. Volume *4*, edited by Denzin NK, Lincoln YS. Thousand oaks, CA: Sage; 2011. 301–316.

[34] DiCicco‐Bloom B, Crabtree BF. The qualitative research interview. Med Educ. 2006;40(4):314–321. doi: 10.1111/j.1365-2929.2006.02418.x. 16573666

[35] Braun V, Clarke V. Using thematic analysis in psychology. Qual Res Psychol. 2006;3(2):77–101. doi: 10.1191/1478088706qp063oa.

[36] Braun V, Clarke V. Conceptual and design thinking for thematic analysis. Qualitative Psychology. 2022;9(1):3–26. doi: 10.1037/qup0000196.

[37] Vaismoradi M, Jones J, Turunen H, et al. Theme development in qualitative content analysis and thematic analysis. 2016.

[38] Lincoln YS, Guba EG. But is it rigorous? Trustworthiness and authenticity in naturalistic evaluation. New Directions for Program Evaluation. 1986;1986(30):73–84. doi: 10.1002/ev.1427.

[39] Denzin NK. Triangulation 2.0*. J Mix Methods Res. 2012;6(2):80–88. doi: 10.1177/1558689812437186.

[40] Tong A, Sainsbury P, Craig J. Consolidated criteria for reporting qualitative research (COREQ): a 32-item checklist for interviews and focus groups. Int J Qual Health Care. 2007;19(6):349–357. doi: 10.1093/intqhc/mzm042. 17872937

[41] World Medical Association. World Medical Association Declaration of Helsinki: ethical principles for medical research involving human subjects. Bull World Health Organ. 2001;79:373. 11357217 PMC2566407

[42] Clark D, Lawton R, Baxter R, et al. Do healthcare professionals work around safety standards, and should we be worried? A scoping review. BMJ Qual Saf. 2025;34(5):317–329. doi: 10.1136/bmjqs-2024-017546. PMC12013549 39332903

[43] Elwyn G, Frosch D, Thomson R, et al. Shared decision making: a model for clinical practice. J Gen Intern Med. 2012;27(10):1361–1367. doi: 10.1007/s11606-012-2077-6. 22618581 PMC3445676

[44] Parekh N, Ali K, Davies K, et al. Can supporting health literacy reduce medication-related harm in older adults? Ther Adv Drug Saf. 2018;9(3):167–170. doi: 10.1177/2042098618754482. 29492245 PMC5810855

[45] Chesser AK, Keene Woods N, Smothers K, et al. Health literacy and older adults: a systematic review. Gerontol Geriatr Med. 2016;2:2333721416630492. doi: 10.1177/2333721416630492. 28138488 PMC5119904

[46] Mor-Anavy S, Lev-Ari S, Levin-Zamir D. Health literacy, primary care health care providers, and communication. Health Lit Res Pract. 2021;5(3):e194–e200. doi: 10.3928/24748307-20210529-01. 34260319 PMC8279021

[47] Pasient- og brukerrettighetsloven. Lov om pasient- og brukerrettigheter [Patient and User Rights Act]. LOV-1999-07-02-63: Lovdata; 1999.

[48] Haukelien H, Møller G, Vike H. Brukermedvirkning i helse-og omsorgssektoren [User participation in the health and care sector]. TF-rapport nr 284. Bø: Telemarksforskning; 2011.

[49] Sammen skal vi redusere risiko og forebygge pasientskader [Together we will reduce risk and prevent patient injuries]. Available from https://pasientsikkerhetsprogrammet.no.

[50] Bikova M, Christensen K. Samproduksjon i mulighetsrommet for brukermedvirkning i norske helse-og omsorgstjenester [Co-production in the space of opportunity for user participation in Norwegian health and care services]. Tidsskrift for Omsorgsforskning. 2022;8(1):1–13.

[51] Riksrevisjonen. Riksrevisjonens Undersøkelse av Tilgjengelighet og Kvalitet i Eldreomsorgen [The Office of the Auditor General’s investigation into the availability and quality of elderly care]. Oslo, Norway: Riksrevisjonen; 2018.

[52] Martín-Oliveros A, Plaza Zamora J, Monaco A, et al. Multidose drug dispensing in community healthcare settings for patients with multimorbidity and polypharmacy. Inquiry. 2024;61:469580241274268. 469580241274268. doi: 10.1177/00469580241274268. 39373170 PMC11526267

[53] Tahvanainen H, Kuitunen S, Holmström A-R, et al. Integrating medication risk management interventions into regular automated dose dispensing service of older home care clients–a systems approach. BMC Geriatr. 2021;21(1):663. doi: 10.1186/s12877-021-02607-x. 34814848 PMC8609790

[54] Rechel B. Hub-and-spoke dispensing models for community pharmacies in Europe. Eurohealth (Lond). 2019;24(4):3–6.

[55] Collier D, Mahoney J. Insights and pitfalls: selection bias in qualitative research. World Pol. 1996;49(1):56–91. doi: 10.1353/wp.1996.0023.

[56] Larson RB. Controlling social desirability bias. International Journal of Market Research. 2019;61(5):534–547. doi: 10.1177/1470785318805305.

