# Supplementary material for: Unseen and unread decision letters in everyday care: a case study of medication management assistance in Norwegian municipal home healthcare
Source: Scand J Prim Health Care. 2026 May 14;44(1):2669814. doi: 10.1080/02813432.2026.2669814 (PMC13178029; doi:10.1080/02813432.2026.2669814)
Supplement: Appendix Case overview.docx [file IPRI_A_2669814_SM2474.docx]

|  |  | *Case 1* | *Case 2* | *Case 3* | *Case 4* | *Case 5* | *Case 6* | *Case 7* | *Case 8* | *Case 9* |
| --- | --- | --- | --- | --- | --- | --- | --- | --- | --- | --- |
| *Decision letter start (which MM services)* |  | *Missing* | *Granted the following assistance: Dosing and controlling medications, administering medications as prescribed.* | *Granted the following assistance: Dosing medications until multidose is implemented. Ensuring medications are taken as prescribed.* | *Granted the following assistance: Administering multidose. Giving medications as prescribed by the physician.* | *Granted the following assistance: Administering and controlling multidose.* | *Help with medication management, the service includes the following: Assistance with installing dose packs in a medication dispenser every other week, help with monitoring medication changes, and picking up medications from the pharmacy when needed.* | *Help with installing dose packs in a medication dispenser every other week, help with monitoring medication changes, and picking up medications from the pharmacy when needed* | *Help with: Administration of medications. Prescriptions. Storage of medications. Preparation and delivery of weekly dose packs.* | *Help with: Ordering medications and prescriptions, storing medications, organizing and checking, and delivering weekly dose packs.* |
| *Decision*  *letter after one year (which MMS services)* |  | *Missing* | *Granted the following assistance: Controlling and administering multidose.* | *Granted the following assistance: Administering medications as prescribed by a doctor. Dosing and monitoring medications.* | *Granted the following assistance: Administering and delivering multidose. Administering medications as prescribed by the physician. Eye drops as prescribed by the physician* | *Granted the following assistance: Administering and monitoring multidose.* | *The service has been changed and now includes the following: Assistance with installing dose packs in a cassette; you insert it yourself into the dispenser. Help with monitoring medication changes and picking up medications from the pharmacy when needed* | *Decision has not been changed (…) Assistance with installing dose packs in a medication dispenser every other week, help with monitoring medication changes, and picking up medications from the pharmacy when needed.* | *No revision* | *Help wit****h:****Ordering medications and prescriptions, storing medications, organizing and monitoring, weekly delivery of dose packs.* |
| *Patient* | *Gender*, age (years old)* | *F, 78* | *M, 82* | *M, 78* | *M, 87* | *F, 90* | *F, 65* | *M, 90* | *M, 70* | *F, 78* |
|  | *living situation* | *alone in a two-story townhouse* | *alone in a semi-detached house alongside his son* | *in a flat, daughter frequently but not permanent staying with him* | *a flat together with spouse* | *alone in a two-story detached house* | *in a two-story detached house with spouse* | *alone in sheltered housing* | *alone in a flat* | *a sheltered housing* |
|  | *work experience* | *Care worker in home healthcare* | *craft business and- teaching* | *repairman for electronics* | *Missing ( not experience from healthcare)* | *Care worker in home healthcare* | *varied professional background (including home service, factory, personal assistant)* | *professional driver, including ambulance* | *experience from electricity company* | *various positions in municipal healthcare* |
| *Nurse* | *Gender*, education* | *F, nurse assistant* | *F,*  *nurse assistant* | *F, nurse assistant* | *F, nurse assistant* | *F,*  *nurse assistant* | *F, nurse* | *F, nurse* | *F, nurse* | *F, nurse* |
|  | *Years of experience* | *22 years of experience in home healthcare and 10 years in nursing home* | *22 years of experience in home healthcare and 10 years in nursing home* | *22 years of experience in home healthcare and 10 years in nursing home* | *14 years of experience in home healthcare* | *22 years of experience in home healthcare and 10 years in nursing home* | *15 years of experience in home healthcare* | *15 years of experience in home healthcare* | *8 years of experience in home healthcare* | *8 years of experience in home healthcare* |
| *Service allocator* | *Gender*, education* | *F, nurse* | *F, nurse* | *F, nurse* | *F, nurse* | *F, nurse* | *F, social educator (and nurse assistant)* | *F, social educator (and nurse assistant)* | *missing* | *missing* |
|  | *Years of experience* | *12 years of experience as purchaser* | *12 years of experience as purchaser* | *12 years of experience as purchaser* | *12 years of experience as purchaser* | *12 years of experience as purchaser* | *30 years of experience from municipal health services* | *30 years of experience from municipal health services* |  |  |
| *General practitioner (gender, experience as GP)* |  | *missing* | *missing* | *F, new in GP role, 30 years as hospital physician, specialist in general medicine, gastrosurgeon* | *missing* | *missing* | *missing* | *missing* | *missing* | *missing* |

*F, female; M, male
